# A CBR-Based and MAHP-Based Customer Value Prediction Model for New Product Development

**DOI:** 10.1155/2014/459765

**Published:** 2014-08-04

**Authors:** Yu-Jie Zhao, Xin-xing Luo, Li Deng

**Affiliations:** Business School, Central South University, Changsha 410083, China

## Abstract

In the fierce market environment, the enterprise which wants to meet customer needs and boost its market profit and share must focus on the new product development. To overcome the limitations of previous research, Chan et al. proposed a dynamic decision support system to predict the customer lifetime value (CLV) for new product development. However, to better meet the customer needs, there are still some deficiencies in their model, so this study proposes a CBR-based and MAHP-based customer value prediction model for a new product (C&M-CVPM). CBR (case based reasoning) can reduce experts' workload and evaluation time, while MAHP (multiplicative analytic hierarchy process) can use actual but average influencing factor's effectiveness in stimulation, and at same time C&M-CVPM uses dynamic customers' transition probability which is more close to reality. This study not only introduces the realization of CBR and MAHP, but also elaborates C&M-CVPM's three main modules. The application of the proposed model is illustrated and confirmed to be sensible and convincing through a stimulation experiment.

## 1. Introduction

Under the furiously competitive market, the competition among similar enterprises has been focused on new product development, that is, a powerful guarantee of the enterprise who wants to enhance the core competitiveness and gain superiority. However, new product development is not only of high cost, but also of higher cost of failure development. Successful new product development is not just a purely technical problem; moreover, it needs to consider various influencing factors. To improve success rate, developers must comprehensively integrate various factors and then correctly evaluate and choose the better development. New product development is a dynamic process that includes raising, analyzing, and solving problems. The decision making, which is affected by wide range factors such as the product's complex structure, is semistructured or even nonstructured. With the maturation of information processing technology and software auxiliary tools, the decision making contributes to not only reducing costs and increasing efficiency, but also improving business strategy [1]. Developers are beginning to make full use of the superiority of software auxiliary tools for better and easier obtaining of new product development. In recent years, many models for new product development have been proposed. However, these highlight the influencing factors including product and market and seldom regard customer needs. Only Chan et al. overcome this shortcoming and propose a new model that integrates three recognized influencing factors of new product development-product attributes specified by designers, customer requirements and satisfaction, and marketing competence. Using system dynamics, the progress for a new product launching the market is stimulated, and the customer lifetime value (CLV) can be obtained. Relative to previous research, Chan's model has incomparable superiority whether on evaluation perspective and method or the influencing factor. But judging from “the basic goal of software engineering” [2], effectively developing software with systematical construction and engineered management, it is obvious that developing systematic software can better meet user needs.

From above perspective, this study finds out three major deficiencies by further analyzing Chan's model. (1) To evaluate the effectiveness of each influencing factor, the experts' workload is too heavy and work time is too long. Meanwhile, the evaluation results have obvious subjectivity deviations. (2) The effectiveness of each influencing factor for different levels of customers is not yet distinguished. (3) Different levels of customers have the same transition probability which is static but dynastic. To overcome the above three deficiencies in Chan's model, this study proposes a CBR-based and MAHP-based customer value prediction model for new product development (C&M-CVPM), and its advantages are as follows. (1) Case based reasoning (CBR) can reduce experts' evaluation workload and work time, which could make up for deficiency one. (2) Multiplicative analytic hierarchy process (MAHP) uses the actual effectiveness of the influencing factor rather than the overall average in stimulation, which could make up for deficiency two. (3) The dynamic customers' transition probability is more close to reality, which could make up for deficiency three.

This section has given the general background to the study.  Section 2 discusses the literature on new product development. The methodology for the proposed C&M-CVPM is presented in Section 3.  Section 4 introduces the model's framework and its three modules. A stimulation experiment is conducted in Section 5. Some concluding remarks are offered in Section 6.

## 2. Literature Review 

### 2.1. The Present State of the Research

In recent years, many conventional and mature models for new product development have been proposed. Xu et al. use fuzzy set and utility and theory to evaluate the typical phases during new product's whole lifecycle [3]; Besharati et al. measure new product development according to the size of customer expected utility, that is, market demand based on customers' preference, designers' experience preference, and uncertainty of product designing characteristics [4]; Alexouda applies algorithm and enumeration method in new product development and then combines the optimal product from the perspective of customer utility maximization and at last returns market share's maximization [5]; Matsatsinis and Siskos discuss market share maximization by historical data (economic or customer survey data) [6]. Hu and Bidanda utilize Markov to build sustainable green product lifecycle's evolution system and then make decisions for new product development from the maximization of the present discount value during the whole product lifecycle [7].

The following three areas, which cover various influencing factors, have been identified as significant and requisite in new product development: (a) product attributes specified by designers [3, 8–10], (b) customer requirements and satisfaction [11–14], and (c) marketing competence [15–18].

However, none of the currently available models considers all of these areas concurrently. Only Chan and Ip [19] integrates three recognized factors and takes the time value of money into consideration, so a new, comprehensive model is proposed on new product development. Within Chan's model, the involved influencing factors are classified into five parts: overall product attractiveness (OPA), overall customer satisfaction (OCS), word of mouth (WOM), marketing approach (MA), and remarketing approach (RA). Based on purchasing frequency, new product customers are divided into five levels: potential customers, first-time customers, regular customers, frequent customers, and loyal customers.

While the system is running, the overall average effectiveness of the above five influencing factor is evaluated by experts or historical data. Then, the customer purchasing model is established by system dynamics and the customer lifetime value (CLV) can be predicted by Markov model. And, at last, this model could evaluate new product development.

### 2.2. Analysis of the Research Status

Relative to previous research, Chan's model has incomparable superiority whether on evaluation perspective and method or the influencing factor. But, in order to better meet user needs, after further analyzing with the evaluation methods used in Chan's model, it can easily find out three specific deficiencies which need to be solved urgently.

*Deficiency (1).* The experts' workload must be reduced by auxiliary software. To be specific, when Chan's model predicts customer lifetime value of new product, the parameters' effectiveness such as production cost is basically obtained with the concerted efforts of experts. However, there is no available data for the product that does not yet enter the market. Therefore, it is hardly feasible to investigate and evaluate those parameters one by one. This is because, firstly, it will impose large workloads to experts and last a long period of time to evaluate each parameter. Secondly, system developers may not have necessarily adequate ability to accurately evaluate each parameter. In addition, the subjectivity from experts could easily lead to certain deviations. 

*Deficiency (2).* For improving the accuracy of evaluation, it is necessary to distinguish the effectiveness of the influencing factors for different levels of customers. Chan thinks that different levels of customers have the same effectiveness of one influencing factor that equals to overall average effectiveness of all levels of customers. However, in real life, the high-frequency purchasing customers often have a relatively high recognition to the product during their purchasing. When the product's overall average effectiveness has reached a certain value, it may not attract the low-frequency purchasing customers, but it can attract the high-frequency ones. 

*Deficiency (3).* Dynamic customers' transition probability is much more realistic. When stimulating the purchasing, Chan uses original nonzero probability as all levels of customers' transition probability. Namely, the level of (*i*) customers' transition probability equals the retained customers' number at time (*i* − 1) divided the customers' number at time (*i* − 2), and it is obvious that the probability is irrelevant to time. So, Chan believes that different levels of customers have the same transition probability that is static but dynastic. However, the real system is filled with dynamics, complexity, and product periodicity. As time passed, each level of customers' transition probability will change within a certain range.

This study takes the advantages of CBR and MAHP and introduces the dynamic transition probability. Based on what is discussed above, C&M-CVPM is proposed. In response to the three deficiencies from Chan's model, this study puts forward three solutions, which are the main contributions and innovations simultaneously. 

*Solution (1)*. CBR in C&M-CVPM helps decision makers to match out the new product which is most similar to the preexisting products, from enterprise's historical data. The preexisting products' parameters are used for the new product. If and only if it lacks historical data, the experts' evaluation is required. 

*Solution (2)*. MAHP in C&M-CVPM distinguishes the effectiveness of each influencing factor for different levels of customers though calculating the influencing weight from experts' preference matrix. 

*Solution (3)*. The dynamic customers' transition probability in C&M-CVPM means that the probability is changing in a certain range, so that the model has a more realistic significance.

## 3. Study Approach 

### 3.1. CBR (Case Based Reasoning)

#### 3.1.1. Overview of CBR

CBR is firstly proposed by Roger Schank in 1982 [20] and has become an important machine-learning method in the field of artificial intelligence [21]. CBR can obtain the solutions of new problems by adjusting solutions of old problems similar to new ones [22]. Cognitive science is the logical origin of CBR's theoretical system [23]. Cognitive science suggests that human can pass on the perceived information to their brains [24] and then brains store and remember the information. So, in the future, if brains encounter with one problem which shares the same or similar stored information, brains can quickly provide the information to solve this problem. As is known to us, this form of human cognition is a common way to solve the problem in reality. And its development inspires and enlightens the artificial intelligence experts and scholars greatly.

Based on the above way of human cognition, CBR uses the knowledge and experience that happened in the accumulated cases to provide reference for those similar cases. Using CBR to deal with the present case is not entirely restarting over again, but it matches the past cases with the present case and modifies the existing methods in order to make the method more suitable to the present case.

**Figure 1:**
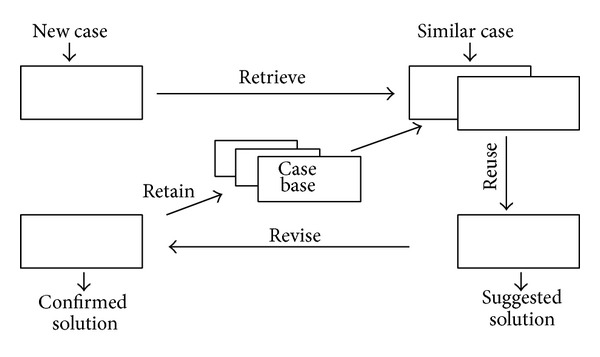
The flowchart of CBR.

Therefore, CBR is more appropriate for those areas such as imperfect knowledge but relatively rich experience. CBR is different from the traditional reasoning method and it can collect the methods resolved the past problems which is the same as or similar to the present one, whereas the traditional reasoning method requires complete domain knowledge. CBR is generally divided into four steps, which are usually called 4R [25]: retrieve, reuse, revise, and retain (see Figure 1). For a new case, CBR will retrieve the same and most similar case from the case vase, reuse the case, and correspondingly revise the suggested solution to get a confirmed solution for the new case. Simultaneously, the amended case will be retained as a new case in the case-base for subsequent uses.

#### 3.1.2. Scope of CBR

Nowadays, CBR has been playing an important role in practical applications because of its advantages, such as the online services applications [26], scheduling and process planning [27, 28], hydraulic mechanical design [29], architectural design [30], customer relationship management [31], troubleshooting [32, 33], design and implementation of knowledge management [34, 35], the prediction of information systems outsourcing success [36], and customer and market plans [37, 38]. CBR has solved many problems successfully. Analyzing and summarizing the findings, CBR is mainly applied in the following cases.

1. The large amount of tacit knowledge stored in experts' brains is not easily extracted. Therefore, when it is not convenient to extract knowledge from vast areas quickly and easily, CBR can straightly extract the valuable tacit knowledge out of the experts' brains to solve problems.
2. When using rule-based reasoning, the amount of tacit rules and professional knowledge stored in experts' brains is huge. So, it is easy to cause combinatorial explosion between each rule and field. Eventually it will lead to reasoning fault and low efficiency. However, CBR can avoid such combinatorial explosion problem.
3. While demanding to quickly update the domain knowledge and rules, CBR can avoid people being involved in the problems of exponential knowledge and information by incremental learning method. And, to a certain extent, CBR can also reduce the problems caused by outdated or incomplete previous experience and knowledge.
4. When facing incomplete past experience, knowledge and rules, or difficult model and structure, such as effective plans of suddenly occurred events, CBR can effectively avoid some problems that are caused by these complex social management or economic system.
5. CBR can be applied in the field of knowledge management which attaches great importance to tacit knowledge. It is far reaching significant for effectively mining the tacit knowledge and making it into explicit knowledge. The knowledge or information carried in the case from CBR is relatively complete fragments, and it includes tacit knowledge to be effectively mined in knowledge management.

#### 3.1.3. Application of CBR in This Study

With aggravated global competition, in order to gain greater competitive advantages, enterprises have to develop new products among the same present products [24]. Belecheanu et al. point out that CBR is more suitable than KBS (knowledge systems) to solve the problem of complex and uncertain new product development [39]. In fact, CBR has been widely used in the product development at present. Therefore, it is feasible to apply CBR in this study.

### 3.2. MAHP

MAHP (multiplicative analytic hierarchy process) is an improvement and extension of AHP (analytic hierarchy process), so it is necessary to do an overview of AHP.

#### 3.2.1. Overview of AHP

In 1970s, Saaty, a famous American operational research expert, proposes analytic hierarchy process (AHP), which divides multicriteria and multiobjective decision making into object, criteria, and project. AHP does quantitative and qualitative analysis on hierarchy. AHP can deeply analyze the nature of complex decision problems, factors, and intrinsic relationships then use minor quantitative information to make thinking progress mathematical and at last provide an easy way for complex decision making mixed of qualitative and quantitative problems. AHP divides relevant elements into goals, guidelines, and programs, and, based on which, it makes qualitative and quantitative analysis decisions [40].

AHP belongs to subjective weighting methods. That is, the decision makers compare pairwise the importance for multiple criteria according to their previous knowledge and experience and then determine the final weight for each criterion from comparison matrix through relevant mathematical model.

#### 3.2.2. Overview of MAHP

Although AHP has been widely used in multiobjective decision, it still has many shortcomings. For example, when using this method, an important step is to check whether the comparison matrix meets the condition of consistency or not, namely, to revise the comparison matrix to ensure the consistency of matrix. However, the consistency always exists in the real world, and this becomes the most fundamental and fatal flaw of AHP. In addition, the presence of incompatibility, incomplete information and reverse caused by the inconsistency of judgment matrix are also the flaws of the AHP.

To solve these problems, in 1990s, Lootsma first proposed a method to improve the original AHP, known as multiplicative analytic hierarchy process (MAHP) [41]. MAHP makes up three decencies in AHP-weight synthesizing, order preserving, and weight scaling. MAHP can improve determination of the weights under various criteria of decision making [42].

#### 3.2.3. Application of MAHP in This Study

Therefore, this study uses MAHP in order to better determine the weights of influencing factors for different levels of customers [1, 43].

## 4. C&M-CVPM 

### 4.1. The System Framework of C&M-CVPM

**Figure 2:**
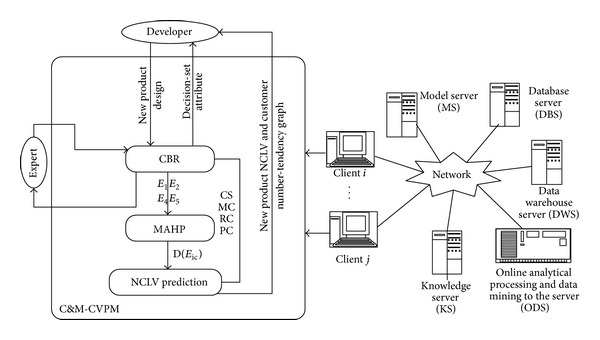
The system framework of C&M-CVPM.

The main functions of C&M-CVPM are providing the customer value for new product development through changing-trend diagram of the net customer lifetime value (NCLV) and the customers' number. The implementation of the functions is divided into three modules: CBR, MAHP, and NCLV prediction module. Each module is running by data or related technical support through model server (MS), knowledge server (KS), database server (DBS), data warehouse server (DWS), and online analytical processing and data mining server (ODS) (see Figure 2).

While C&M-CVPM is running, CBR module could work out the decision-set attributes for the preexisting product which is most similar to the new product and then input these parameters as the new product's corresponding parameters (*E*_1_, *E*_2_, *E*_4_, and *E*_5_) to MAHP module. The decision-set attributes include each parameter and developing experience. The development can adjust, reevaluate, and also modify the inappropriate attributes to gain more satisfying results. The development experience is the lessons learned from the preexisting product, marketing, or remarketing experience. In addition, if the matched similarity is lower than the threshold set by experts during running CBR module, C&M-CVPM will ask for experts' assistance to provide the value of those parameters to be evaluated through experts' investigation or information provided from the relevant servers.

C&M-CVPM uses the integrated construction on network [44]; each server uses both C/S (client/server) mode, on which the resources can be shared and services can be provided to other severs or different clients simultaneously.

C&M-CVPM's users include the product developer, who is the actual operator of the system, the domain experts, who are responsible for stabling and maintaining the sharing resources on all kinds of servers, such as the knowledge in KS and the models in MS, and the system administrators, who should be duty bound to maintain the whole system from the information technology perspective.

The clients correspond to the integrated components of traditional decision support system, the system for problem synthesis and interaction. Firstly, the new produce development is inputted into the clients, and then the relevant system control program is generated; at last all types of servers through the network are called to evaluate the new product development. Meanwhile, the developer can also query the shared resources on all types of serves to meet their own needs. MS, KS, DBS, DWS, and ODS are responsible for related data and technical support.

Database server (DBS) updates single user's database and database management systems and adds network protocol, communication protocols, concurrency control, security mechanisms, and other server functions, mainly for storing all source data of enterprise's production, sales and research, and so on. DBS provides support for DWS.

Data warehouse server (DWS) is composed of warehouse management system (WMS), data warehouse (DW), and analysis tools. For better centralizing and simplifying part work of DWS, and also for more efficiently achieving the function of DWS, analysis tools here do not include online analytical processing and data mining. Data warehouse mainly stores the data from DBS by the extracting, transforming, and loading. The data set is subject-oriented, integrated, stable, and at different time, including historical data of enterprise's all products, current data, and integrated data. DWS offers support for ODS.

Online analytical processing and data mining server (ODS) is independent of DWS. During its runtime, a large amount of data extract from DWS to ODS builds a temporary warehouse; then ODS can find out a deeper level of assistant decision information. ODS is mainly responsible for updating and maintaining preexisting products' information, finding the relationship between the loss rate of customers (LR) and overall customer satisfaction (OCS) and the relationship between word of mouth (WM) and OCS. ODS offers support for MS.

Model server (MS), based on single user's model base and model-base management system, increases network protocol, communication protocols, security mechanisms, and other server functions. MS mainly stores all mathematical models and data processing models necessary for C&M-CVPM's three modules; mathematical models includes all the algorithms and equations such as the similarity calculation equations in CBR, while data processing models are used for selecting, sorting, and summarizing the data. The models are implemented during MS runtime; meanwhile the data, information, and knowledge on other severs can also be called up as required.

Knowledge server (KS) upgrades single user's knowledge base management system, knowledge base, and inference engine and furthermore adds network protocol, communication protocols, concurrency control, security mechanisms, and so forth. KS is generally responsible for storing a large number of production rule knowledge and fact knowledge such as the threshold in CBR of matched similarity.

### 4.2. The CBR Module

As discussed in the introduction, CBR in C&M-CVPM can help decision makers to match out the new product case, which is most similar to the preexisting product cases, from historical enterprise's data [45]. The preexisting product's parameters are able to use for the new product. In Chan's model, these parameters can be acquired only by experts' evaluation, including CS (the cost of the goods), MC (the marketing cost), RC (remarketing cost), PC (the number of potential customers), RP (the retail price of the product), *E*_1_ (overall product attractiveness), *E*_2_ (overall customer satisfaction), *E*_4_ (marketing effectiveness), and *E*_5_ (remarketing effectiveness).

The key techniques in CBR are the indexation for case representation and the similarity evaluation [46] and can efficiently match out the most similar preexisting case from the case-base with the new product. Therefore, from these two aspects, the section below will illustrate how CBR helps C&M-CVPM obtain the above parameters to be predicted.

#### 4.2.1. Case Representation

**Figure 3:**
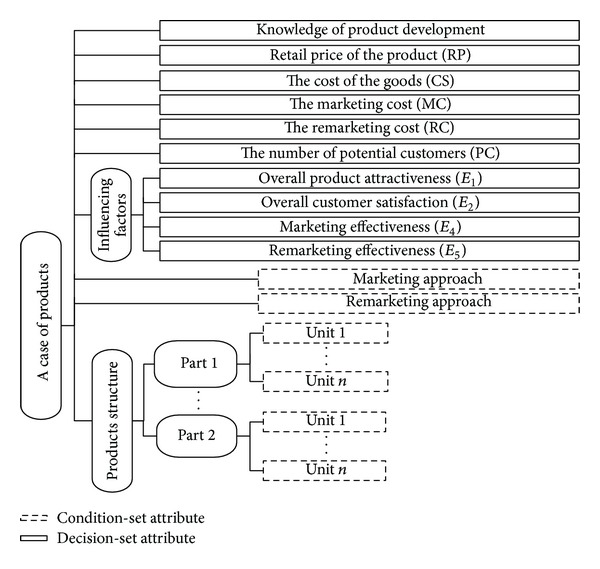
The frame representation results for the product case.

There are many approaches for case representation and the knowledge expression method in artificial intelligence. An overwhelming majority of cases in intelligent case-base use the frame representation. In reality, identification of different products can be done by their structural features (including specific functions, appearance, and quality and implementation techniques). So, the frame representation can obviously better meet the product's characteristics. Because the frame representation expresses the same thoughts and ideas with CBR, this study uses the framework representation to express the product case in CBR module (see Figure 3). The dotted boxes represent the condition-set attributes, and the solid boxes represent the decision-set attributes (i.e., the parameters to be predicted). CBR will automatically work out the similarity of the condition-set attributes between new and preexisting products and then output the decision-set attributes which have maximum similarity as the corresponding parameters for new product.

#### 4.2.2. Similarity Evaluation

There are many algorithms to evaluate the similarity of the product's condition-set attributes. Gu et al. [47] propose the FRAWO which has obvious advantages over the traditional approaches in retrieval efficiency and the quality of retrieval results. Therefore, making use of FRAWO, C&M-CVPM can realize similarity evaluation between the products. Specific processes are as follows.

Preexisting product cases are stored in the product case-base. Assuming that the product case-base has *m* cases, the case (*i*)—*X*_*i*_ has *n* attributes values such as *X*_*i*1_, *X*_*i*2_,…, *X*_*in*_, and the values of *n* condition-set attributes for the target new product case *G* are *G*_1_, *G*_2_,…, *G*_*n*_; then the condition-set attributes matrix *D*(*X*_*ij*_) can be obtained by (1). And the average of each column of *D*(*X*_*ij*_) is calculated by (2), with the intermediate variable being (3):

(1) D(Xij) =[XG] =[X11X12⋯X1nXl1Xl2⋯Xln⋮⋮⋱⋮Xm1Xm2⋯XmnG1G2⋯Gn]︷n  condition − set  attributesm  cases  in  case  base + target  case  G

(2) $$\begin{array}{c} \bar{C_{j}}=\frac{\sum_{i=1}^{m+1}X_{ij}}{m+1}, \end{array}$$

(3) $$\begin{array}{c} M_{ij}=\frac{X_{ij-}\bar{C_{j}}}{C_{j}}. \end{array}$$

The normalized effectiveness matrix *D*(*X*_*ij*_′) is expressed in (4), and *X*_*ij*_′ is stated in (5):

(4) $$\begin{array}{c} D\left(X_{ij}^{\prime }\right)=\left[\begin{array}{c} X_{i}^{\prime }\\ G^{\prime } \end{array}\right] \end{array}$$

(5) $$\begin{array}{c} X_{ij}^{\prime }=\frac{1-\exp\left(-M_{ij}\right)}{1+\exp\left(-M_{ij}\right)}\;X_{ij}^{\prime }\in \left[-1,1\right] \end{array}$$

The similarity between the preexisting product case (*i*) and the target new product case is determined by (6):

(6) sim(Xi,G)=sim(Xi′,G′)=1−dis(Xi′,G′)=1−∑j=1nwj·d(Xij′,Gj′)d(Xij′,Gj′)=1−sim(Xij′,Gj′)sim(Xij′,Gj′)={{1,Xij′=Gj′0,Xij′≠Gj′,when  Xij′  and  Gj′  are  symbol  propertiesexp⁡(−|Xij′−Gj′|max⁡(i)−min⁡(i)),when  Xij′  and  Gj′  are  certainly  numeric  propertiesa(Xij′∩Gj′)a(Xij′)+a(Gj′)−a(Xij′∩Gj′),when  Xij′  and  Gj′  fuzzy  properties.

*a* stands for the surface of corresponding subordinate functions, and *a*(*X*_*ij*_′∩*G*_*j*_′) stands for the intersection of two fuzzy surfaces.

*w*_*j*_ is the matched weight of the similarity for the condition-set attributes, and its initial value is set by experts based on their experience. When using the corresponding parameters for new product, the strategy for adjusting the weight is for those preexisting product cases whose similarity exceeds the threshold set by experts; if the C&M-CVPM's predictive result is correct, the system must increase the weights of condition-set attributes which are of the same value as the target new product case, and every increscent is Δ*i*/*k*_*c*_. *k*_*c*_ stands for the times that the preexisting product case is properly matched, while Δ*i* is the adjustment range of weights set by experts' experience. Of course, Δ*i* can also be adjusted as actual needs.

### 4.3. MAHP Module

As mentioned earlier, MAHP in C&M-CVPM can calculate the weight of some influencing factors for different levels of customers through experts' preference matrix and then work out the gap between the effectiveness of this influencing factor and overall average effectiveness. Finally the effectiveness of this influencing factor for different levels of customers can be obtained. The specific procedures are as follows.

The overall average effectiveness of these five influencing factors for new product is set as *E*_1_ (overall product attractiveness, OPA), *E*_2_ (overall customer satisfaction, OCS), *E*_3_ (word of mouth, WOM), *E*_4_ (marketing effectiveness, MA), and *E*_5_ (remarketing effectiveness, RA). The weight for the influencing factor (*c*) for level (*i*) customers is expressed as *w*_*i*_, where *i* = 1,2,…, 5 represents potential, first-time, regular, frequent, and loyal customers and *c* = 1,2,…, 5 represents the five influencing factors: OPA, OCS, WOM, MA, and RA. Totally *k* experts participate in the evaluation, according to Table 1, the expert (*p*)  (*p* = 1, 2 …, *k*) should determine the influencing gap *δ*_*ij*_^(*p*)^ between level (*i*) and level (*j*) customers of the influencing factor (*c*).

The preference matrix for the expert (*p*) is expressed in (7), and the relationship between *δ*_*ij*_^(*p*)^ and the weights *w*_*ic*_^(*p*)^ and *w*_*jc*_^(*p*)^ is stated in (8). The constrain is determined by (9). Consider

(7) $$\begin{array}{c} D_{c}^{\left(p\right)}={\left(\delta_{ij}^{\left(p\right)}\right)}_{c}, \end{array}$$

(8) $$\begin{array}{c} \frac{w_{ic}^{\left(p\right)}}{w_{jc}^{\left(p\right)}}=e^{-\ln\sqrt{2}\delta_{ij}^{\left(p\right)}}, \end{array}$$

(9) $$\begin{array}{c} \overset{5}{\underset{i=1}{\prod }}w_{ic}^{\left(p\right)}=1. \end{array}$$

Using a logarithmic least squares estimation, the influencing weight from the expert (*p*) for level (*i*) customers can be predicted by (10). The influencing weight of all *k* experts for level (*i*) customers is expressed mathematically by (11). By normalization, the weight of the influencing factor (*c*) for level (*i*) customers is determined by (12). Consider

(10) $$\begin{array}{c} w_{ic}^{\left(p\right)}=\exp\left(\frac{\ln\sqrt{2}}{5}\overset{5}{\underset{j=1}{\sum }}\delta_{ij}^{\left(p\right)}\right), \end{array}$$

(11) $$\begin{array}{c} w_{ic}^{*}=\exp\left(\frac{\ln\sqrt{2}}{5k}\overset{k}{\underset{p=1}{\sum }}\;\overset{5}{\underset{i=1}{\sum }}\delta_{{}_{ij}}^{{}^{\left(p\right)}}\right) \end{array}$$

(12) $$\begin{array}{c} w_{ic}=\frac{w_{ic}^{*}}{\sum_{j=1}^{5}w_{jc}^{*}},\;i=1,2,\ldots ,5. \end{array}$$

As in Chan's model, different levels of customers have the same effectiveness of the influencing factor, and every level's effectiveness of the influencing factor (*c*) is *E*_*c*_/5. After dividing by MAHP, the allocated effectiveness of the influencing factor (*c*) for level (*i*) customers is *w*_*ic*_ · *E*_*c*_. Therefore, the gap of between the effectiveness of the influencing factor (*c*) and the overall average of level (*i*) customers is stated in (13). If Δ*E*_*ic*_ < 0, the effectiveness does not reach the overall average; if Δ*E*_*ic*_ > 0, it exceeds the overall average; if Δ*E*_*ic*_ = 0, it exactly equates to the overall average. *D*(*E*_*ic*_) stands for the effectiveness matrix to each influencing factor for different levels of customers, and then *E*_*ic*_ is easy to get by (14):

(13) $$\begin{array}{c} \Delta E_{ic}=w_{ic}\cdot E_{c}-\frac{E_{c}}{5}, \end{array}$$

(14) $$\begin{array}{c} E_{ic}=E_{c}+\Delta E_{ic}. \end{array}$$

### 4.4. The Predictive Module for NCLV

**Figure 4:**
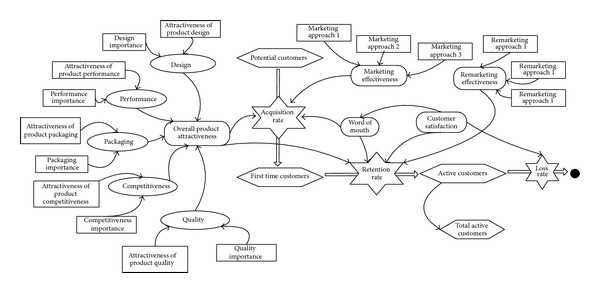
The stimulation figure of customer purchasing.

The main functions of this module are to predict the new product customer value by stimulating customer purchasing and inputting the parameters; those are the output of CBR and MAHP modules (see Figure 4). The parameters to be stimulated include CS, MC, RC, PC, RP, and *D*(*E*_*ic*_).

The stimulation of customer purchasing, used in the predictive module for NCLV (net customer lifetime value), has the same basic idea in line with Chan's model proposed by system dynamics. But when calculating CLV (customer lifetime value) by Markov model, the randomly dynamic customers' transition probability replaces the static probability. Correspondingly, the relationships between some nodes are adjusted appropriately for better logicality, understandability, and practicability. The following is the specific process of the simulation.

Following Chan's study, NCLV (net customer lifetime value) is defined as the sum of the current lifetime values of all customers, where customers lifetime value refers to the present value of future profit from a customer.

As mentioned earlier, new product's customers are divided into five levels: potential, first-time, regular, frequent, and loyal customers. In Chan's model, the second to the fourth level customer is regarded as a whole, that is, active customer; the customers' increase is derived from the second level. Within Chan's formula, the customers' retention, which refers to the increment from the second level to the third, fourth, and fifth level, used the numbers of the third, fourth, and the fifth level from the input port. But apparently, for one specific level, the increase in purchasing frequency and transiting quantity to next level must be affected by the level itself rather than the next level. Hence, on one hand, this study follows the calculation equations in Chan's model to randomly obtain the “retention rate” (RR) and the “acquisition rate” (AR) (see (16)). On the other hand, the relationships and customers number must be made appropriate adjustments.

**Figure 5:**
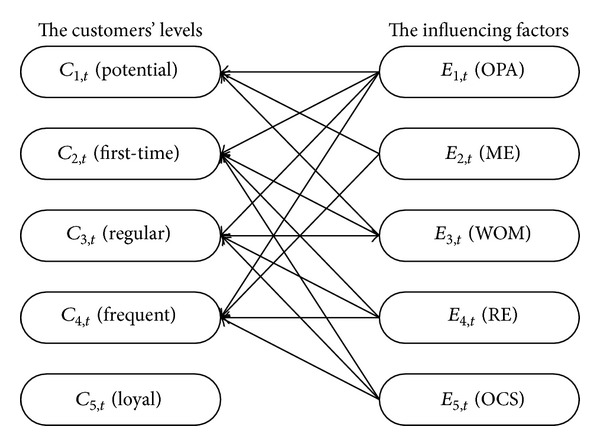
The relationships between customers' levels and influencing factors.

Obviously, the influence factors for different customer's levels are not the same. The potential customers of the product make purchasing on the basis of OPA, ME, and WOM. The first-time and regular customers are influenced by OPA, WOM, RE, and OCS. The frequent customers are affected by OPA, RE, and OCS. However, the loyal customers only trust enterprise's certain product because of their complete allegiance and will not look for other alternatives. So in general the loyal customers are not affected by any factors (see Figure 5).

**Figure 6:**
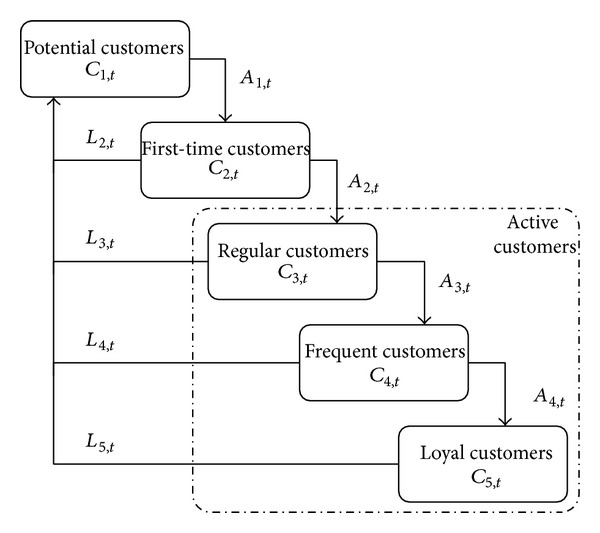
The relationship between different levels.

In C&M-CVPM, the customer purchasing behavior for new product is divided into three statues. The first statue is that the customers will no longer buy this new product because of dissatisfaction; thereby, the first level customers mean the lost ones who will become the potential customers. The second statue is that the customers, who are very satisfied with the new product, will increase purchasing frequency for the next time and thus come into the next level. The third statue is that the customers, who neither like nor dislike the new product, will keep the former purchasing frequency. Most notably, the lost customers, who are not satisfied with this new product, do not mean that they will never buy it in future; they may be affected by other various factors and purchase this new product again. The relationship between different levels is shown as in Figure 6.

*C*_*s*,*t*_ denotes the number of customers in level (*s*) at time (*t*); AR_*s*,*t*_ means the acquisition rate of the customers who transit to the next level in level (*s*) at time (*t*); *A*_*s*,*t*_ indicates the transition number of customers in level (*s*) at time (*t*); LR_*s*,*t*_ implies the loss rate of customers in level (*s*) at time (*t*); *L*_*s*,*t*_ describes the transition number of customers in level (*s*) at time (*t*). The relationships between *C*_*s*,*t*_, AR_*s*,*t*_, LR_*s*,*t*_, and *L*_*s*,*t*_ are expressed in (15):

(15) $$\begin{array}{c} C_{s,t}\times \text{A}{\text{R}}_{s,t}=A_{s,t},\\ C_{s,t}\times \text{L}{\text{R}}_{s,t}=L_{s,t}. \end{array}$$

**Figure 7:**
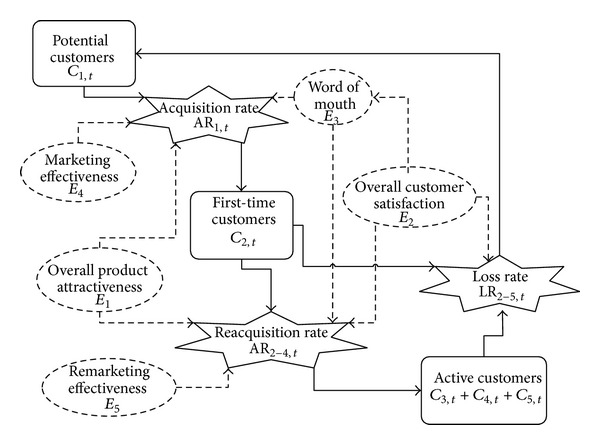
The relationship between influencing factors and AR or LR.

The relationships between the five influencing factors, the “acquisition rate” (AR), and the “loss rate” (LR) can be observed from Figure 7.

It is easy to know that word of mouth (*E*_3_) and LR are mainly decided by overall customer satisfaction (*E*_2_). Therefore, the relationships, between *E*_2_ and *E*_3_, LR and *E*_2_, can be acquired through data mining of historical product. So, once the value of *E*_2_ is worked out by CBR similarity matching, then the value of *E*_3_ and LR could be generated automatically. The relationships between the unity matrix *D*(*E*_*ic*_) and the acquisition rate (AR) are expressed in (16):

(16) AR1,t=random{min⁡(E11,E13,E14),max⁡(E11,E13,E14)},AR2,t=random{min⁡(E21,E22,E23,E25),    max⁡⁡(E21,E22,E23,E25)},AR3,t=random{min⁡(E31,E32,E33,E35),max⁡⁡(E31,E32,E33,E35)},AR4,t=random{min⁡(E41,E42,E43,E45),max⁡⁡(E41,E42,E43,E45)}.

Assumptions are as follows: one, customers buy new products under noncontractual relationship. That is to say, customers purchasing is entirely based on their own preferences; two, all customers make purchasing decisions at the same time point, and the time interval (*d*_*t*_) is equivalent.

Hence, *C*_*s*,*t*_, the number of customers at level (*s*) at time (*t*), are determined by (17):

(17) C1,t={C1,t−dt+(L2,t−dt+L3,t−dt+L4,t−dt    + L5,t−dt−A1,t−dt),t≠0PC,t=0C2,t={C2,t−dt+(A1,t−dt−L2,t−dt−A2,t−dt),t≠00,t=0C3,t={C3,t−dt+(A2,t−dt−L3,t−dt−A3,t−dt),t≠00,t=0C4,t={C4,t−dt+(A3,t−dt−L4,t−dt−A4,t−dt),t≠00,t=0C5,t={C5,t−dt+(A4,t−dt−L5,t−dt),t≠00,t=0.

Set *D* as the current discount rate, by the randomly dynamic transition probability; (18) is applied to calculate the NCLV:

(18) NCLV=(∑t=0∞[C2,t×(RP−MC−CS)   +(C3,t+C4,t)×(RP−RC−CS)   +C5,t×(RP−CS)])×((1+D)t)−1.

### 4.5. C&M-CVPM's Characteristics and Application Scope

#### 4.5.1. C&M-CVPM's Characteristics

To overcome the above three shortcomings in Chan's model, this study proposes a CBR-based and MAHP-based customer value prediction model for new product development (C&M-CVPM). The following are the new model's characteristics.

1. C&M-CVPM evaluates the quality of new product development by using the net customer lifetime value (NCLV), which is in line with the customer-oriented market tendency. Therefore, it has an advantage over formers studies in the prediction perspective.
2. C&M-CVPM considers the time value of profits bringing by customers. Hence, it would work out the net present customer value of new product development.
3. C&M-CVPM stimulates the customer purchasing behavior with fully considering all the three recognized influencing factors for new product development, which are product, customer, and market.
4. C&M-CVPM reduces the experts' workload by using CBR so that removes the unnecessary evaluation of each influencing factor on each product.
5. C&M-CVPM shortens the time to predict the customer value for new product development, simultaneously improving the evaluation's agility.
6. C&M-CVPM, by using MAHP, distinguishes the effectiveness of each influencing factor for different levels of customers.
7. C&M-CVPM uses dynamic customers' transition probability to simulate customer purchasing behavior, which can make the model more realistic and authenticity.
8. C&M-CVPM, by using CBR, avoids the same mistake in the developing process as low as possible, and it can also promote the retention and heritance for enterprises' tacit knowledge to prevent possible losses caused by a mature technology leaving the company. Also, C&M-CVPM can provide references for the new product development which is conducive to the existence and improvement of development level.
9. C&M-CVPM takes the marketing cost into account to evaluate the customer value, and this is also helpful for knowledge exchanging and sharing between enterprises design and marketing department.
10. C&M-CVPM expands the system users' scope so that the ordinarily nonexpert developer could also predict the new product's customer value to better satisfy user needs.
11. C&M-CVPM, by using CBR, can solve the new problems as human thinking mode with the aid of the formerly accumulated knowledge and experience. Basically, it can eliminate bottlenecks in retrieval due to knowledge and information index growth; furthermore the previous case is relatively easy to be collected.

#### 4.5.2. The Application Scope of C&M-CVPM

Although C&M-CVPM has many advantages over former studies, there are still some limitations for the application scope of C&M-CVPM.

1. There is a premised assumption for CBR applying to C&M-CVPM; that is, similar problems have similar solutions. So, new product development to be predicted must have certain similarity with the cases in case-base. For example, food products in case-base cannot be used to predict commodity products. And it may not make sense even if the case to be predicted and the case in case-base belong to one same type. For example, if predicting electric toothbrush by ordinary toothbrush, the effect may not be ideal, and vice versa.
2. The new product customers are divided into five levels: potential, first-time, regular, frequent, and loyal customers. However, this division may not be applied to some products, such as household appliances that the customer will not buy another in the short term. So, the new product customers might be divided into two or three levels as well. At the same time, those products which own five-level customers usually belong to FMCG (fast moving consumer goods).
3. If the case-base has insufficient product cases, the predictive results may be not accurate enough, so C&M-CVPM is more suitable for those enterprises that already have a lot of mature products.
4. From the foregoing, sufficient mature cases provide a strong guarantee for the good predictive results. Consequently, building the original case-base is a huge project that needs to consume certain human, material, and financial resources. Enterprise's decision whether to use C&M-CVPM or not is depending on its own situation.
5. CBR, based on the incremental learning, has an automatic-learning mechanism with the formerly accumulated knowledge and experience. But seen in another way, because of unconditionally passive learning, each reserved case in the case-base may easily lead to an unmanageable state. Correspondingly, the system will run with a low efficiency and the retrieval cost is going up. In conclusion, CBR should try active learning in the face of a large number of samples.
6. The Markov model that is applied to calculate customer lifetime value in C&M-CVPM is the most flexible model within the present studies, but it still constitutes some limitations. For example, during the transaction between customers and enterprises, each interval time is same and fixed.

## 5. Simulation Experiment and Analysis

In this section, to verify C&M-CVPM's validity, a simulation experiment is conducted to predict the NCLV for some regular toothbrush. The computer auxiliary software is MATLAB R2010a. Firstly, this section describes the reasons why we choose regular toothbrush as the simulation case. Secondly it gives out the experiment's general design, including the purposes, preparations, and producers. At last, it analyzes the experiment's results in detail.

### 5.1. The Simulation Case

In order to make the experiment more intuitive and understandable, and according to C&M-CVPM's assumption, a new regular toothbrush is chosen as our experimental case. Here are the reasons: (1) to some extent, toothbrush has become the necessities of life; (2) toothbrush belongs to FMCG (fast moving consumer goods) because of its regular replacement; (3) there are two main types in the market, regular and electric toothbrush, and regular toothbrush gains relatively larger market share due to the price factor and people's habits.

### 5.2. The Experiment's General Design

#### 5.2.1. The Experiment's Purpose

C&M-CVPM's first major advantage lies in reducing the experts' workload during new product development. With the introduction of CBR, the parameters of the running system are decided by the most similar historical product's data automatically provided by system, rather than totally depending on experts' evaluation. If and only if the similarity is less than experts' setting threshold, experts just do the evaluation. The above advantage, combining with the latter two advantages, improves the accuracy of the system. Thus, verifying the validity of C&M-CVPM is equal to verifying the reduction of experts' workload and the increment of system accuracy compared to Chan's model.

However, on the other hand, the accuracy for the system proposed by Chan is strongly subjective because it is more likely to rely on the experts' ability. In reality, the accuracy of the running system may be different when one expert predicts different products or different experts predict one product. Therefore, one or a few experiments are meaningless and far from enough and it cannot distinguish the good development from the bad. For another hand, when running C&M-CVPM, the system accuracy can be verified very well with comparing the predictive value and the actual value for product customer value.

The purpose of this experiment is to test that (a) C&M-CVPM can reduce the experts' workload when predicting new product customer value for a regular toothbrush and that (b) the deviation from the predictive to the actual value can be acceptable in reasonable range.

#### 5.2.2. The Experiment's Preparations

*Experiment Tool*. This study uses MATLAB that has strong processing power and ease of use as computer auxiliary tool to verify the C&M-CVPM's validity.

**Figure 8:**
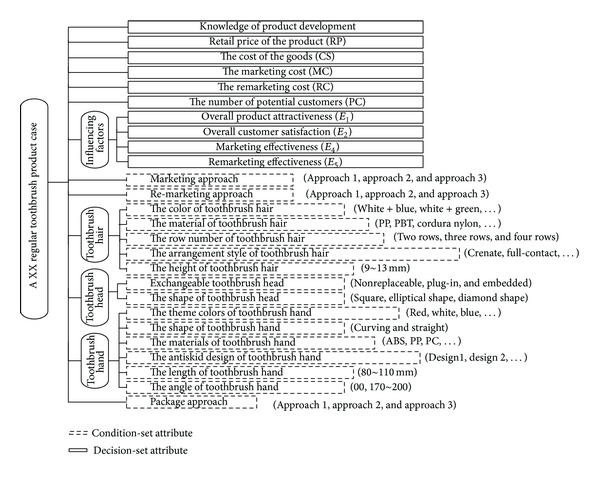
The frame representation of a regular toothbrush.

*Case Representation*. According to product's structural features of the regular toothbrush, the frame representation can be expressed as Figure 8.

*Data Preparation*. CBR is an automatic machine-learning technology, and it can get the overall average of new product's influencing factors and stimulate other running models' parameters by analyzing the inherent rule from historical products' data, so the experiment results seldom rely on authentication of the product data.

According to product's structural features and their inherent relationships, this experiment randomly generates 70 toothbrush cases as the classic case-base. In order to enhance the result's trustworthiness, the study does strict validity design. Except randomly generating experimental data, the data for automatic learning is separated from the data for testing the C&M-CVPM's running accuracy. 60 cases of the 70 classic cases are served as a case-base (enterprise's preexisting products), and the other 10 cases are served as test case (new products). 10 cases as a batch and the case-base can be constructed by inputting six batches, and each batch must do once validity verification after the input is complete.

For the sake of the experiment objectivity, this study invites three professors and two doctors from Business School of Central South University as the expert team. They compare all levels of customers' importance of each influencing factor and then provide 25 importance comparison tables. From those tables, the effectiveness matrix *D*(*E*_*ic*_) can be got.

Things to note are as follows.

1. The initial value of matching weight for each condition-set attribute's similarity is set to 1/*n*, where *n* refers to the number of product cases' condition-set attributes; namely, each condition-set attribute has equal contribution to similarity evaluation at the beginning.
2. To adjust the weight to the best as soon as possible, the weight adjustment Δ*i* is set to 0.02 for the first three batches and 0.01 for the last three batches.
3. The cases are increasing along with more and more experiments, and the weights of each decision-set value have constant adjustment. The later on the C&M-CVPM going, the larger similarity matched by CBR module is. In order to increase the validity for each experiment comparing results, the detected threshold is set to 0.4 for the first three experiments and 0.85 for the last three experiments.
4. The accuracy of C&M-CVPM depends on the NCLV deviation rate of the predictive result for new product test case. Because C&M-CVPM has used the most similar case in case-base as the test case, the NCLV deviation rate measures how far the predictive NCLV value from the actual value for the test case is.
5. The criteria based on experience to judge the consistency of similarity matched results are that the NCLV deviation rate is 3% at most.
6. *d*_*t*_ is set to 3 months, because the dentist advises that toothbrush should be changed every three months. And the potential customers' number is set to 8 million.
7. Per unit of experts' workload equals to the experts workload to predict the parameters, CS, MC, RC, PC, RP, *E*_1_, *E*_2_, *E*_4_, and *E*_5_, when running the Chan's model one time.

### 5.3. The Experiment's Procedures

1. 10 test cases are numbered sequentially from 1 to 10, and six batches for constructing the case-base are numbered sequentially from 1 to 6.
2. The initial value of matching weight for each condition-set attribute's similarity in CBR module is set to 1/*n*, where *n* refers to the number of product cases' condition-set attributes.
3. The expert team is responsible for comparing all levels of customers' importance of each influencing factors and then provides their preference matrix. Finally the effectiveness matrix *D*(*E*_*ic*_) can be calculated by (8)–(10).
4. The first batch cases are inputted into case-base while doing the first experiment. For each inputted case, the matching weight of similarity during each condition-set attribute in CBR module must be adjusted once, where Δ*i* is set to 0.2.
5. After inputting 10 test cases, the following data must be recorded for each case: (a) the highest similarity after each test case matching all the cases, (b) the predictive NCLV deviation rate for each case, (c) the number of new produce development by C&M-CVPM's prediction with the deviation rate limited to 5% most, and (d) the experts' workload units in C&M-CVPM when the cases in case-base are not sufficient.
6. The average for NCLV deviation rate refers to the reduced percentage for the experts' workload. Assume that the experts' workload in Chan's model is 10 units for 10 text cases, so the reduced percentage = (10 − the experts' workload units in one C&M-CVPM experiment)/10.
7. Inputting the next batch, the matching weight of similarity during each condition-set attribute in CBR module must be adjusted once, where Δ*i* is set to 0.2 for the former three experiments and 0.1 for the latter ones. Repeat steps (4)–(6) to finish all the six experiments.

### 5.4. The Experiment's Results

#### 5.4.1. The Maximum Similarity

**Figure 9:**
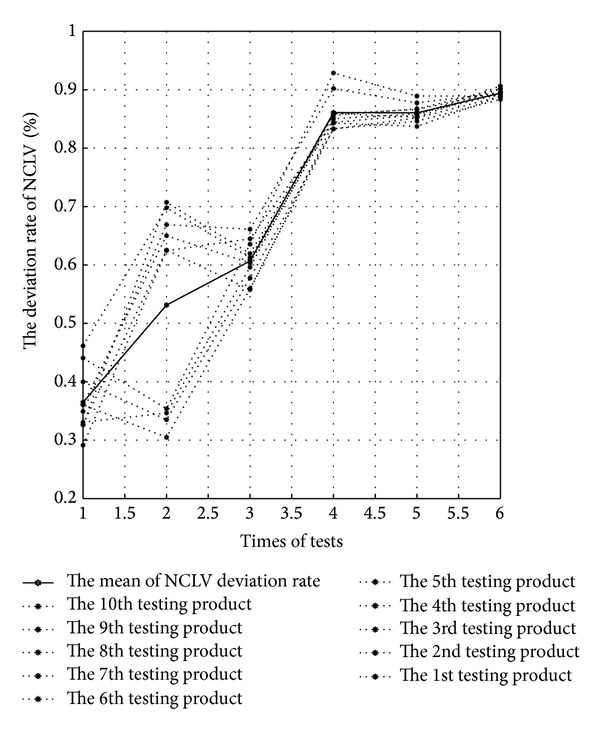
The similarity comparison results of six tests.

The similarity comparison results of six tests are shown in Figure 9. The abscissa represents experiment sequence, and the ordinate represents the value of the similarity. The points on the dotted lines mean the maximum similarity after each test case matching all the cases, and the points on the solid lines mean the average for all the maximum similarity.

Along with the increasing cases, the matching weight of similarity during each condition-set attribute in CBR module has been constantly adjusted and obviously remains rising. When Δ*i* is fixed, the increase range is progressively decreasing for both the former three experiments and the latter three. More specifically, the increase range for the third and the sixth experiment is inconspicuous. The result is further evidence of the validity that Δ*i* is adjusted from 0.2 to 0.1. The maximum similarity for the sixth experiment trends toward 0.9, and it illustrates that a certain amount of cases has been included in the case-base. On one hand, each case could retrieve the case very similar to itself; on the other hand, 60 cases selected in the case-base are feasible.

#### 5.4.2. The NCLV Deviation Rate

**Figure 10:**
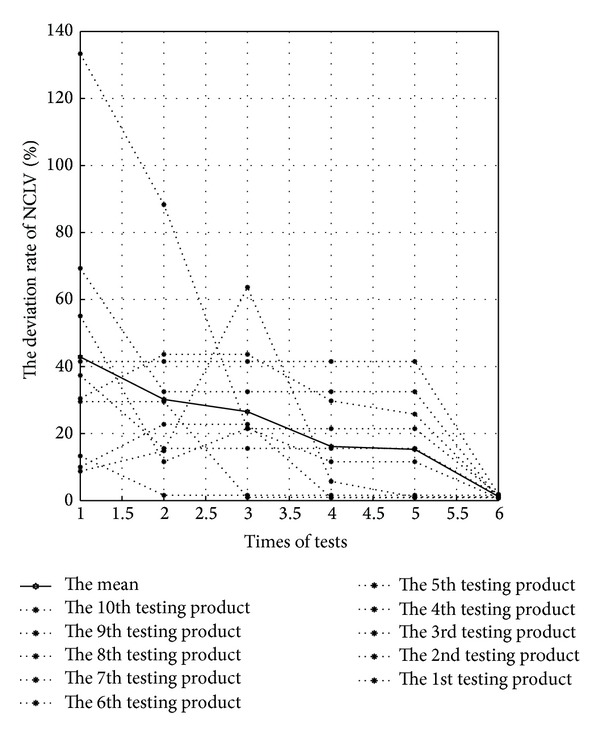
The comparison results of the NCLV deviation rate of six tests.

In Figure 10, the abscissa represents experiment sequence and the ordinate represents the NCLV deviation rate. The points on dotted lines mean the ratio from the NCLV actual value to the predictive value for each test case in C&M-CVPM's every experiment, and the points on solid lines mean the average for overall NCLV deviation rates.

The cases are increasing along with more and more experiments, but the NCLV deviation rate remains declining obviously, as well as the average of overall NCLV deviation rates which are slowly falling and going to 0 at last. For one thing, it demonstrates that six experiments are feasible; for another, C&M-CVPM's capability to correctly predict test cases' NCLV is progressively enhancing. Hence, C&M-CVPM can always better play its unique advantage in predicting new produce development for those enterprises that have owned plenty of mature products.

#### 5.4.3. The C&M-CVPM's Validity

From a qualitative angle (see Figures 9 and 10), C&M-CVPM's capability to match out the most similar and accurate case is gradually strengthened, with the cases' incensement and the similarity's matching weight adjustment. From a quantitative angle, Table 2 can better illustrate the above advantage by the related data.

Along with continues case number's growth and the weight's adjustment, even the detected thresholds for the latter three experiments clearly improve better than the former three; C&M-CVPM's ability to effectively reduce the experts' workload and actually predict customer value is still improving (see Table 2). It is also suggested that C&M-CVPM always plays its unique advantage in predicting new produce development for those enterprises that have owned plenty of mature products.

**Figure 11:**
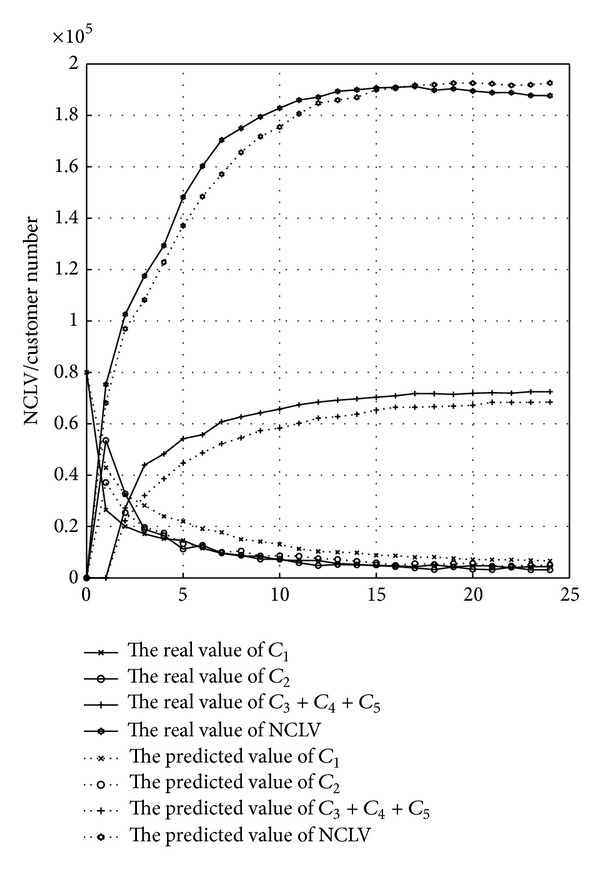
The comparison results for the test case with maximum similarity.

**Figure 12:**
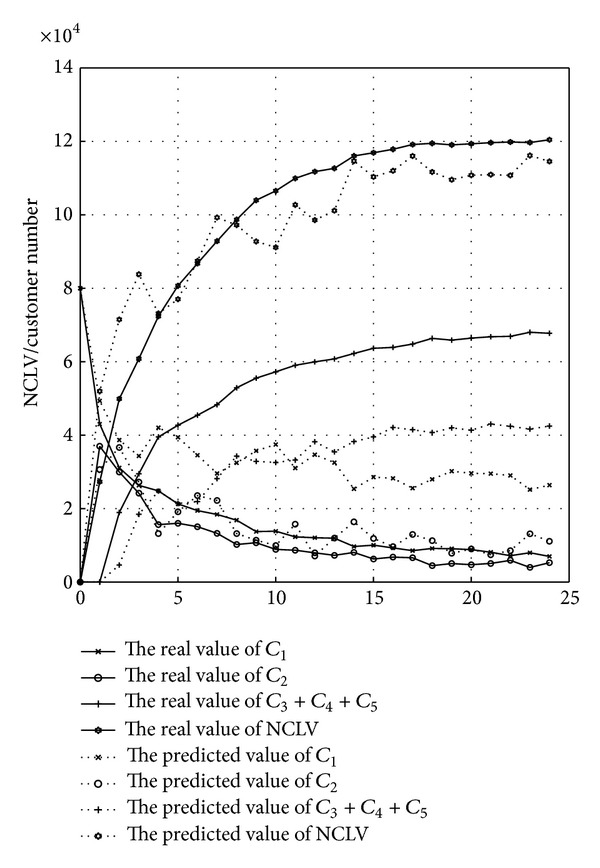
The comparison results for the test case with minimum similarity.

To further clarify the accuracy that C&M-CVPM can predict the new product customer value, from all six experiments, this study chooses the 4th test case with the maximum similarity and the 10th test case with the minimum similarity and then compares their NCLV's predictive and actual values (see Figures 11 and 12). The dotted lines mean the predictive number of all levels customers and the solid lines mean the actual number.

This study compares the NCLV predictive value with the actual values only for the first 24 time points (3 months as a time point and 24 points equal to 6 years). The comparison results for only 24 time points can be more intuitive, and furthermore it is not need to focus on the NCLV's value during all product life cycle. From Figures 11 and 12, it is easy to know that, although there is a small deviation in predicting customers' number, it is still accurate in predicting NCLV's value. That is, C&M-CVPM's ability to actually predict customer value for new product development can be well-trusted.

#### 5.4.4. Other Results

**Figure 13:**
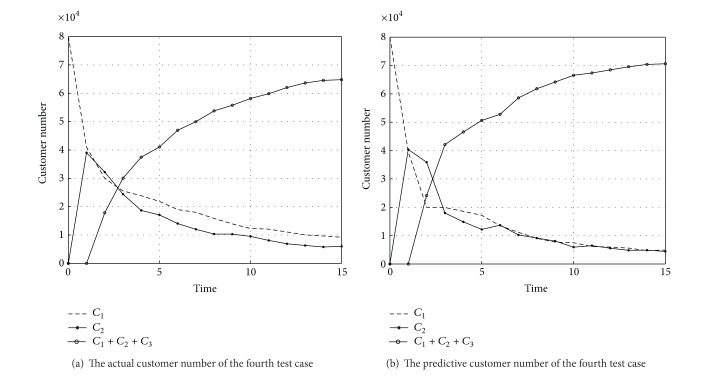
The tendency figure of the actual and predictive customers number of the test case (4).

Because of limited samples and simulated experiments, the accuracy to predictive customers' number in C&M-CVPM is not obvious at present. But, with after further analysis of Figure 13, the potential customer is active and the first-time customer is rapidly increasing when the previous year for selling new product. Moreover, both of them start falling one year later. Consequently, enterprises must lay stress on marketing competence for new product which can obtain much more customer value. More specifically, it is more suitable for enterprises to do advertisement, sales exhibition, internet marketing, and so on. While, one year later, the first-time customers start to reduce along with that the active customers begin to increase. Namely, enterprise strategy should pay more focus on retaining customers and provide more remarketing activities such as affiliate campaigns.

Besides, Figure 13 also makes clear that the active customers' number has reached maximum at the 15th time point (around the 3.75th year). This indicates that the target market is already saturated, which means that customers will not increase their purchase frequency and be not attracted anymore; therefore, seldom new potential customers will come in. In such a situation, enterprises should develop more new products to attract and retain customers. On the other hand, this is also the reason why constant developing new product is the key of enterprise's core competence.

### 5.5. The Experiment's Summary

Notwithstanding, if this study can get a lot of specific enterprises' historical data and compare the accuracy with Chan's model many times, the validity of C&M-CVPM can be verified more thorough. But under the condition of the limited human, material, and financial resources, the randomly generated data to verify the C&M-CVPM's validity is feasible in fact.

Also, this study can be treated as a periodically fundamental work on the current condition. Here are the two reasons: (a) the system running results seldom rely on the data's authenticity because CBR can automatically obtain the values of the influencing factors through historical data and (b) in order to enhance the result's trustworthiness, the study does strict validity design, not only randomly generating experiment data according to the product features, but also separating the data for automatic learning from the data used for testing the running accuracy of C&M-CVPM.

## 6. Conclusions 

To sum up, better than Chan's model, C&M-CVPM distinguishes the effectiveness of the influencing factors for different levels of customers and thinks about the random variation in a certain range and meanwhile reduces the experts' workload to investigate and evaluate the required parameters with the lack of historical data for new product's customer value. Besides, better than Chan's model, C&M-CVPM has the following four functions: (a) it expands the system users' scope so that the ordinarily nonexpert developers can also evaluate the new product's customer value. Meanwhile, the same mistakes can be possibly prevented from happening to improve the accuracy of problem solution; (b) it promotes the retention and heritance for enterprise's tacit knowledge; (c) it outputs the similar product development's experience for decision-maker reference which can help to enhance the developers skill and also gather enterprise marketing team's knowledge to promote information sharing; (d) it eliminates bottlenecks for knowledge retrieval.

In conclusion, superior to Chan's model, C&M-CVPM can better satisfy user needs and urge the decision progress more scientification, procedures, and automation.

The purpose of C&M-CVPM as a periodically fundamental work has been achieved. Future research could gather larger and more complex practical samples. Meanwhile, more work is needed to obtain the specific enterprise's historical data to test the validity of C&M-CVPM and compare repetitiously with Chan's model. However, the divisions of five levels customers in C&M-CVPM may not apply to some products expect FMCG (fast moving consumer goods). The Markov model in C&M-CVPM, which is the most flexible model within the present models, used to calculate customer lifetime value still constitutes some limitation such as that each interval time is same and fixed.

## Figures and Tables

**Table 1:** The influencing gap *δ*_*ij*_^(*p*)^ on different levels of customers.

*δ*_*ij*_^(*p*)^	Definition
0	Influence of factor (*c*) on customer (*i*) is the same as customer (*j*)
2	Influence of factor (*c*) on customer (*i*) is a little bigger than customer (*j*)
4	Influence of factor (*c*) on customer (*i*) is a bit bigger than customer (*j*)
6	Influence of factor (*c*) on customer (*i*) is much bigger than customer (*j*)
8	Influence of factor (*c*) on customer (*i*) is rather bigger than customer (*j*)

**Table 2:** The verified results of the validity of C&M-CVPM.

Tests	The threshold	The products number	Experts' evaluation time	The reduction for experts' workload (%)	The average of overall NCLV deviation rate (%)
1	0.4	2	8	20	42.32
2	0.4	6	4	60	27.82
3	0.4	10	0	100	26.52
4	0.85	6	4	60	18.15
5	0.85	8	2	80	15.69
6	0.85	10	0	100	1.21
